# Immunomodulation Mediated by Anti-angiogenic Therapy Improves CD8 T Cell Immunity Against Experimental Glioma

**DOI:** 10.3389/fonc.2018.00320

**Published:** 2018-08-20

**Authors:** Courtney S. Malo, Roman H. Khadka, Katayoun Ayasoufi, Fang Jin, Jackson E. AbouChehade, Michael J. Hansen, Raymond Iezzi, Kevin D. Pavelko, Aaron J. Johnson

**Affiliations:** ^1^Department of Immunology, Mayo Clinic, Rochester, MN, United States; ^2^Mayo Clinic Graduate School of Biomedical Sciences, Mayo Clinic, Rochester, MN, United States; ^3^Department of Ophthalmology, Mayo Clinic, Rochester, MN, United States; ^4^Department of Neurology, Mayo Clinic, Rochester, MN, United States; ^5^Department of Molecular Medicine, Mayo Clinic, Rochester, MN, United States

**Keywords:** glioblastoma, anti-angiogenic therapy, immunotherapy, vaccine, combination therapy

## Abstract

Glioblastoma (GBM) is a lethal cancer of the central nervous system with a median survival rate of 15 months with treatment. Thus, there is a critical need to develop novel therapies for GBM. Immunotherapy is emerging as a promising therapeutic strategy. However, current therapies for GBM, in particular anti-angiogenic therapies that block vascular endothelial growth factor (VEGF), may have undefined consequences on the efficacy of immunotherapy. While this treatment is primarily prescribed to reduce tumor vascularization, multiple immune cell types also express VEGF receptors, including the most potent antigen-presenting cell, the dendritic cell (DC). Therefore, we assessed the role of anti-VEGF therapy in modifying DC function. We found that VEGF blockade results in a more mature DC phenotype in the brain, as demonstrated by an increase in the expression of the co-stimulatory molecules B7-1, B7-2, and MHC II. Furthermore, we observed reduced levels of the exhaustion markers PD-1 and Tim-3 on brain-infiltrating CD8 T cells, indicating improved functionality. Thus, anti-angiogenic therapy has the potential to be used in conjunction with and enhance immunotherapy for GBM.

## Introduction

Glioblastoma (GBM) is a lethal cancer of the central nervous system (CNS). Patients diagnosed with GBM have a median expected survival of about 15 months following diagnosis with treatment (1, 2). As it currently stands, there is no cure for GBM, and even with surgical resection of the tumor, a patient will universally recur and succumb to disease. Therefore, there is a clear need for the development of new therapies for GBM treatment.

One such therapeutic strategy that has been rising in popularity are immunotherapies, which aim to target the immune system to respond to the tumor. Immunotherapies provide a facet of precision not possible with surgical techniques, which are unable to target the invasive edges of the tumor, or chemotherapies, which nonspecifically target all dividing cells (3). As a result, numerous research groups are testing a variety of immunotherapy strategies against GBM tumors, both in pre-clinical models and in clinical trials (2). In particular, strategies to activate tumor antigen-specific CD8 T cells, which will then kill tumor cells using cytotoxic granules, have been promising (4). While these therapies have demonstrated some success, there are still no curative strategies for GBM. This is primarily due to the immune suppressive nature of the tumor microenvironment, and the global immune dysregulation patients present with despite immunotherapy treatments.

To simultaneously bypass the immune suppressive tumor environment and stimulate anti-tumor immune responses, concomitant therapies have become highly prevalent. These treatment regimens often combine a therapy that is currently in use with a novel immunotherapy, including vaccination (2). Importantly, the synergy between many of these combination treatments has not been defined. For example, combining anti-angiogenic therapies, often used in patients with recurrent GBM following surgical resection, with immunotherapies, improves survival in pre-clinical models (5). However, the extent to which anti-angiogenic therapy blocking vascular endothelial growth factor (VEGF) impacts the immune response to GBM directly, is unclear. Studies in other tumor models and in *in vitro* assays have suggested a regulatory role of VEGF on the immune system (6, 7). These studies in particular demonstrate a role for VEGF on retention of dendritic cells, a potent antigen presenting cell (APC), in state of reduced activation. This would in turn reduce T cell activation and subsequently negate the impact T cell-based immunotherapy strategies, including tumor antigen-specific vaccination.

We hypothesized that blockade of VEGF using the clinically available anti-angiogenic therapy, VEGF-Trap (Eylea/Aflibercept), we would improve dendritic cell maturation and in turn improve antitumor T cell responses in a murine model of GBM, the GL261-quad cassette syngeneic glioma. Our group has previously demonstrated that treatment with VEGF-Trap, which is a VEGF receptor (VEGFR) fusion protein conjugated to a human IgG Fc region, results in similar outcomes as GBM patients treated with bevacizumab anti-angiogenic therapies as measured by T1- and T2-weighted magnetic resonance imaging (MRI) and histology (5). Likewise, VEGF-trap treatment improves survival in GL261-quad cassette bearing animals (5). Importantly, VEGF-Trap is used in place of bevacizumab due to improved cross-reactivity with murine VEGF (8).

To address this hypothesis, we first assessed the expression of VEGFRs on the surface of dendritic cells, which we contend are the most potent APC to generate CD8 T cell responses in the CNS (9). We also treated GL261-quad cassette bearing animals with VEGF-Trap weekly and assessed the quality of dendritic cell activation in the tumor draining lymph nodes (TLDNs) 14 days post treatment. We also evaluated the proportion of tumor antigen-specific CD8 T cells in the CNS of these animals.

## Materials and methods

### Acute viral infection and vaccination

Six- to eight-week-old C57BL/6 mice were infected intracranially (i.c.) with Theiler's murine encephalomyelitis virus (TMEV) as previously described (9–11). Animals were anesthetized with 1–2% isoflurane, then received a single dose of 2 × 10^5^ plaque forming units (PFU) of TMEV in the right hemisphere of the brain. VEGFR expression was measured in the draining lymph nodes and brain 5 and 7 days following infection.

### GL261 cell culture and implantation

The GL261-quad cassette cell line has been transgenically modified to express four model antigens: OVA_257−264_, OVA_323−339_, human GP100_25−33_, and ${\text{I-E}}_{52-68}^{\text{a}}$, in addition to a luciferase transgene to assess tumor burden. 6 × 10^4^ GL261-quad cassette cells were implanted by stereotactic injection as previously described (5, 10). Six- to eight-week-old female C57BL/6 animals were anesthetized with 20 mg/kg ketamine and 5 mg/kg xylazine to minimize discomfort during the procedure. Cells were injected at a concentration of 6 × 10^4^ GL261 cells per 1 μL phosphate buffered saline (PBS). Injection rate was 0.2 μL per minute. The site of injection was 1 mm lateral, 2 mm anterior of the bregma with a depth of 3 mm from the surface. All animal experiments were approved by and performed in accordance with the Mayo Clinic Institutional Animal Care and Use Committee.

### Bioluminescence imaging

GL261-quad cassette-bearing animals were assessed for tumor burden using bioluminescence imaging as previously described (5, 10). Animals were intraperitoneally injected with 150 mg/kg D-luciferin sodium salt in PBS (Gold Biotechnology, Olivette, MO). Animals were anesthetized with 1–2% isoflurane before and throughout imaging. Animals were scanned using an IVIS Spectrum system (Xenogen Corp., Amameda, CA, USA) running Living Image software. Bioluminescence intensity (photons/s) was recorded in a circular region of interest surrounding the head. Animals with average bioluminescence intensity above 10^5^ photons/s were considered tumor bearing and treated with VEGF-Trap or PBS. All animal work was completed in accordance to the Mayo Clinic Institutional Animal Care and Use Committee guidelines.

### Anti-angiogenic therapy treatment

VEGF-Trap/Aflibercept (Regeneron Pharmaceuticals, Rensselaer, NY, USA) was administered at a dose of 12.5 mg/kg in PBS in a total volume of 100 μL intravenously (i.v.) by injection into the tail vein 2 weeks post-tumor injection. Treatment was continued weekly until animals were euthanized for flow cytometry analysis. Control mice received 100 μL PBS i.v., at the same time points.

### Flow cytometry

Lymph nodes and spleens were harvested in RPMI and pressed through a 70 μm filter to achieve a single cell suspension for compensation control samples. Brains were harvested and manually homogenized using a dounce homogenizer as previously described (12). Brain samples were filtered through a 70 μm filter to achieve a single cell suspension into a 50% percoll solution. Samples were centrifuged at 7,840 g. The myelin debris layer formed at the top of the gradient was aspirated. All samples were washed twice and plated in a 96-well v bottom plate. Peptide:MHC tetramers were constructed by our lab and samples were stained at a 1:100 dilution of tetramer for 30 min at room temperature in the dark. Following a wash, antibodies against CD45, CD11c, CD11b, I-A^b^, CD80, CD86, VEGFR2, Nrp-1, CD4, CD8α, PD-1, and Tim-3 were used for staining at a 1:100 dilution for 30 min on ice in the dark (BD Biosciences, San Jose, CA; Tonbo Biosciences, San Diego, CA) in addition to Ghost Red 780 Viability Dye used at a 1:1000 dilution (Tonbo Biosciences, San Diego, CA). Samples were fixed with 2% paraformaldehyde. Samples were subsequently run on a BD LSRII flow cytometer equipped with FACSDiva software (BD Biosciences, San Jose, CA). Samples were digitally compensated using single-stained controls and analyzed by FlowJo v10 software (FlowJo LLC, Ashland, OR).

### Statistical analysis

All data are presented as mean ± standard error of the mean (SEM). Significance was determined using a Mann-Whitney Rank Sum Test. GraphPad Prism 7.0 (La Jolla, CA) were used for all statistical analysis.

### Data availability

All data generated during this study are available from the corresponding author on reasonable request.

## Results

### Dendritic cells express VEGFRs in the inflamed CNS

**Figure 1:**
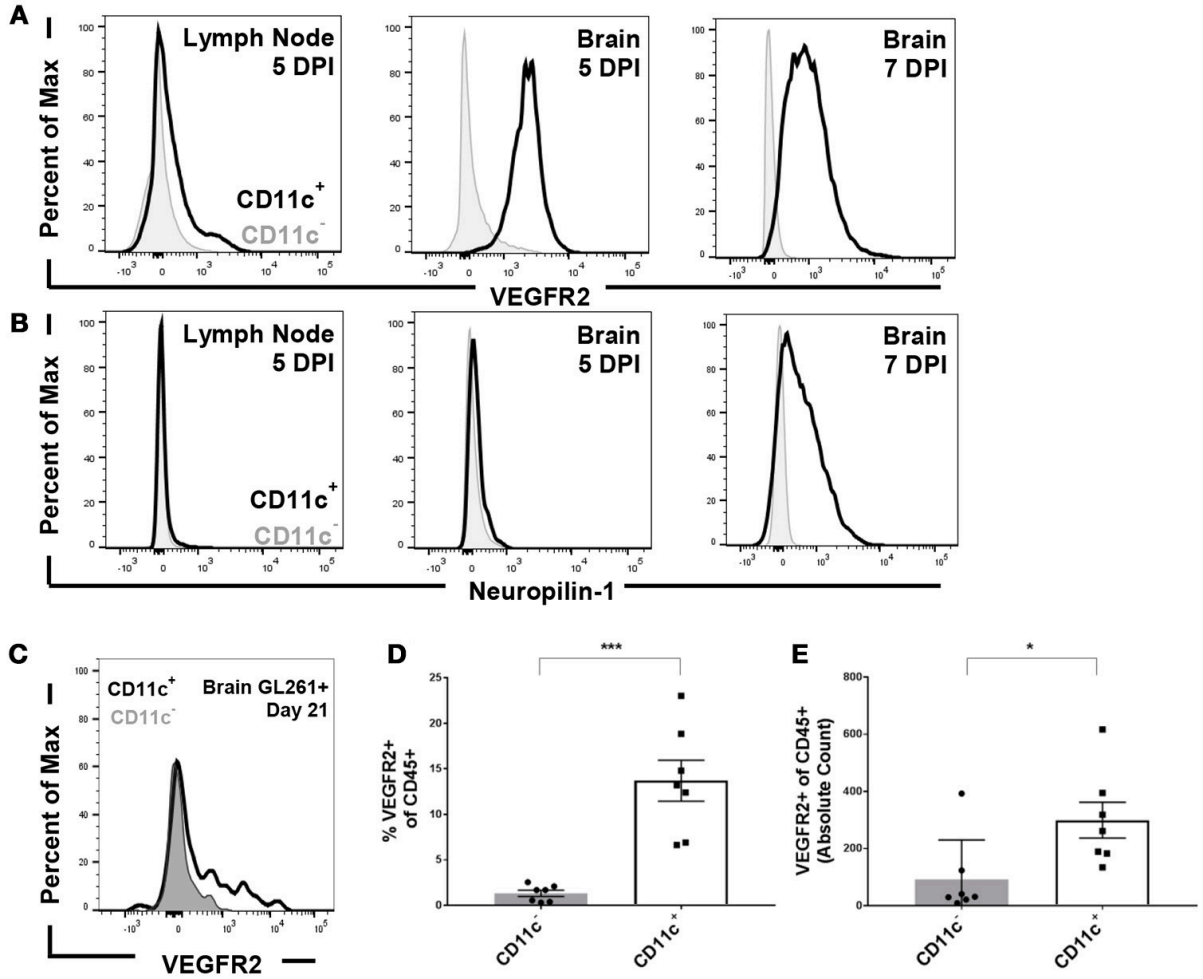
Dendritic cells express VEGFR2 and neuropilin-1 in the brain during picornavirus infection and 21 day established GL261 glioma. Dendritic cells were isolated from the cervical lymph node and brain five and seven DPI. Dendritic cells were gated as CD45^hi^ and CD11c^+^. **(A)** Dendritic cells in the lymph node and brain express VEGFR2, with a majority of dendritic cells expressing VEGFR2 seven DPI in the brain. **(B)** Brain-infiltrating dendritic cells express neuropilin-1, a co-receptor for VEGF, in the brain seven DPI with TMEV. **(C)** Representative flow plot showing expression of VEGFR2 in both CD11c^+^ and CD11c^−^ cells isolated from the brain of unvaccinated animal bearing GL261 glioma. **(D,E)** In untreated mice with 21 day established GL261 gliomas, we observed CD11c+ dendritic cells in the brain express higher levels of VEGFR2 (*N* = 7). Data presented as mean with error bars representing standard error of the mean (SEM). *p ≤ 0.05 and ***p ≤ 0.001 by Mann–Whitney *U*-Test.

To address the impact of VEGF signaling on the immune system, we first sought to identify the cells through which VEGF would signal. To address this question, we used infection with Theiler's Murine Encephalomyelitis Virus (TMEV) as a model of CNS inflammation. Intracranial infection with TMEV results in extensive immune cell expansion in the deep cervical lymph nodes and subsequent immune cell infiltration into the CNS (13). We therefore assessed VEGFR expression on CD11c^+^ dendritic cells, compared to CD11c^−^ immune cells, 5 and 7 days post infection. Expression of VEGFR2, considered the primary signaling receptor for VEGF, and Neuropilin-1, known as a co-receptor for VEGF signaling. We found that CD11c^+^ dendritic cells express low but detectable levels of VEGFR2 in the deep cervical lymph nodes 5 days post infection, and express higher levels of VEGFR2 in the brain 5 days post infection (Figures 1A,B). By 7 days post infection, CD11c^+^ cells in the brain express high levels of both VEGFR2 and neuropilin-1, suggesting that dendritic cells in the CNS are capable of signaling through VEGF receptors (Figures 1A,B). This also suggests that neuropilin-1 expression is induced following inflammation. Notably, we do not see upregulation of VEGFR2 or neuropilin-1 expression on CD11c^−^ immune cells.

We next evaluated VEGFR expression on antigen presenting cells (APCs) in the brain in unvaccinated C57BL/6 mice harboring established GL261 gliomas. We primarily focused on dendritic cells owing to its dominant role in mounting anti-glioma response (9). We determined that dendritic cells isolated from the brain of these animals expressed VEGFR2 at higher levels than CD45+, CD11c^−^ blood derived cell types (Figure 1C). Furthermore, the proportion and absolute counts of isolated dendritic cells that express VEGFR2 is significantly higher in glioma bearing mice (Figures 1D,E). We further assessed the expression levels of VEGFRs on other brain-infiltrating and resident immune cells as well. We found the VEGFR2 levels on CD45^int^ CD11b^+^ microglial cells remained unchanged in comparison to non-tumor bearing littermates (data not shown). Similarly, the expression level on other CD45+, CD11c- immune cell types was unremarkable (Figures 1C–E).

### Dendritic cells are more activated, and CD8 T cells are less exhausted, following anti-angiogenic therapy

After demonstrating that dendritic cells express VEGFRs, we next sought to determine the impact of this expression on anti-glioma immune responses. To accomplish this, we implanted GL261-quad cassette gliomas in C57BL/6 mice. Two weeks following tumor implantation, we imaged animals using bioluminescence imaging to remove animals from the study that did not bear tumors. We treated only tumor-bearing animals with VEGF-Trap intravenously. A second cohort of animals was treated with PBS as a control. Two weeks following treatment, or 4 weeks post-tumor implantation, brains were harvested and processed for flow cytometric analysis.

**Figure 2:**
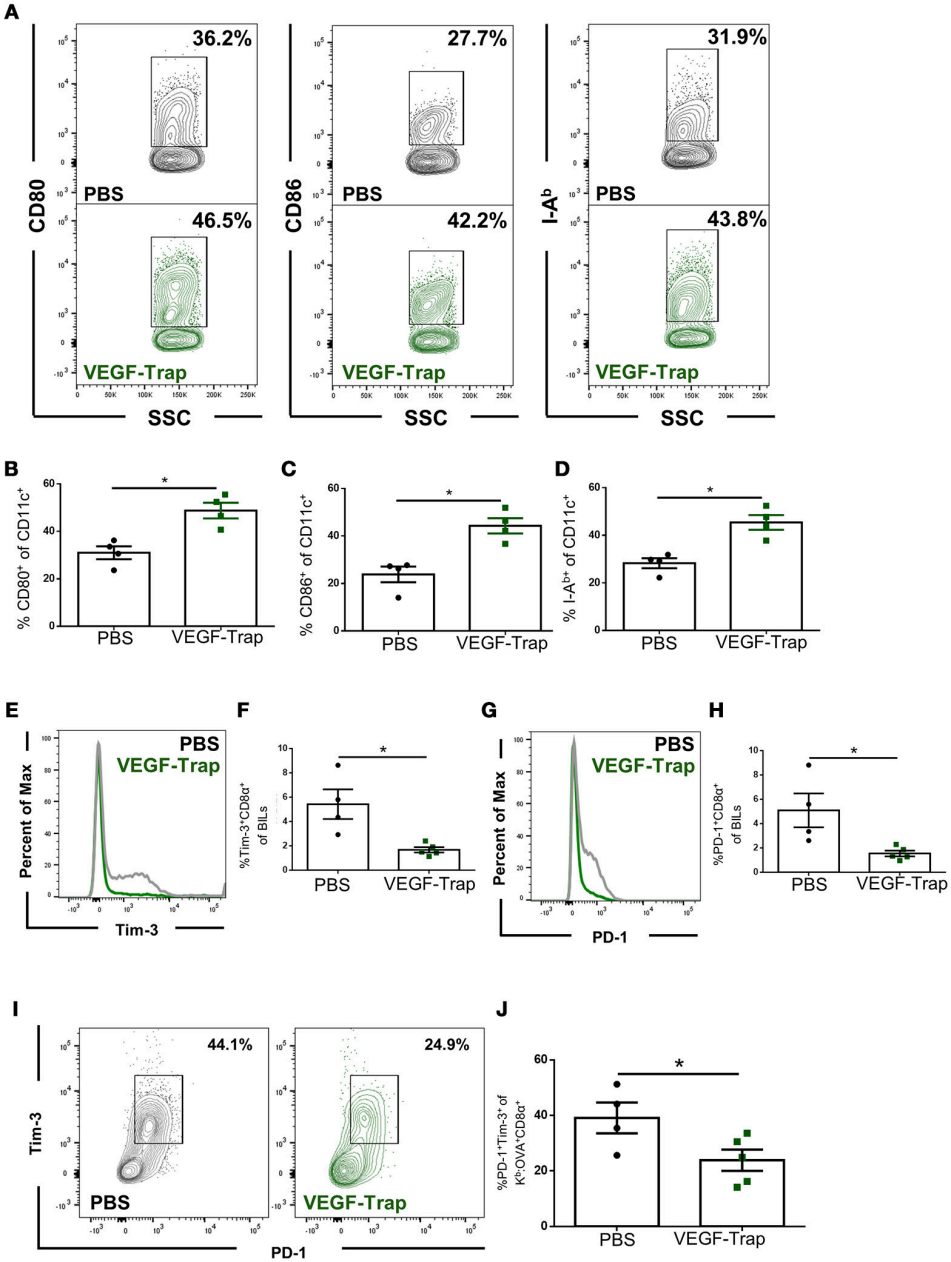
CD8 T cells and dendritic cells isolated from the brain of VEGF-Trap treated GL261-quad cassette bearing mice express a more functional phenotype. GL261-quad cassette bearing animals were treated with PBS (*N* = 4) or VEGF-Trap (*N* = 5) 2 weeks post-tumor implantation. Animals were sacrificed 30 days after tumor implantation and brain infiltrating leukocytes (BILs) were assessed by flow cytometry. **(A)** Representative images of CD11c^+^ cells isolated from the brain assessing expression of costimulatory markers. VEGF-Trap treatment results in increased expression of CD80 **(B)**, CD86 **(C)**, and I-A^b^ MHC Class II **(D)**. Representative flow plots **(E)** and quantification **(F)** show reduction in the proportion of Tim-3^+^ CD8 T cells in the CNS of GL261-quad cassette bearing animals. Representative flow plots **(G)** and quantification **(H)** show a reduction in the proportion of CD8 T cells expressing PD-1 in the brain. A reduction in PD-1^+^Tim-3^+^ double positive CD8 T cells was also observed **(I,J)**. Error bars represent mean ± SEM. *p < 0.05. Side Scatter (SSC) was included as a measure of granularity.

Dendritic cells isolated from the brains of tumor bearing animals were assessed for expression of known activation markers, including CD80 (B7-1), CD86 (B7-2), and I-A^b^ major histocompatibility complex (MHC) class II. We found that following VEGF-Trap treatment, a higher proportion of dendritic cells expressed each of these markers, as compared with PBS treatment (Figures 2A–D). These markers are required for T cell activation, and increase in each of these markers suggests that VEGF-Trap treatment results in dendritic cells that are better capable of stimulating an anti-tumor immune response.

We next assessed the impact VEGF-Trap treatment had on brain infiltrating, tumor antigen-specific CD8 T cells. To accomplish this, we measured expression of the exhaustion markers PD-1 and Tim-3 (14). We assessed proportion of cells expressing these markers on both total CD8 T cells and on K^b^: OVA-specific CD8 T cells, as the GL261-quad cassette cell line expresses OVA peptide (SIINFEKL) as a model tumor antigen (10). We determined that fewer CD8 T cells infiltrating the brain following VEGF-Trap treatment had high expression of PD-1 and Tim-3 (Figures 2E–H). Therefore, a reduced proportion of CD8 T cells are exhausted as a result of VEGF-Trap treatment. Furthermore, tumor antigen-specific CD8 T cells, defined as being Kb:OVA Tetramer^+^, are also less exhausted than tumor antigen-specific CD8 cells isolated from PBS treated animals (Figures 2I,J). These findings suggest that VEGF-Trap treatment results in a tumor-specific CD8 T cell response that is more capable of carrying out their cytotoxic effector function.

## Discussion

Here we demonstrate that VEGF-Trap treatment, as one example of anti-angiogenic therapy, results in a treatment response beyond vasculature normalization. In addition to the previously demonstrated effects observed by this treatment in the GL261 glioma model, we observe a significant change in dendritic cell maturation status and in CD8 T cell exhaustion. These findings are of great importance as immunotherapies are developed for CNS cancers.

Dendritic cell maturation is key for effective antigen presentation of tumor antigens. This is true for both generation of an endogenous immune response as well as in the context of vaccination. We demonstrate that dendritic cells isolated from the lymph nodes of VEGF-Trap treated animals exhibit enhanced expression of costimulatory molecules such as CD80, CD86, and MHC class II. Therefore, dendritic cells from VEGF-Trap treated animals have the capacity to be better antigen presenting cells. Likewise, CD8 T cells isolated from VEGF-Trap treated animals have a demonstrable decrease in exhaustion markers. CD8 T cell exhaustion has been shown to be mediated by the immune suppressive tumor microenvironment, and VEGF is likely one way this is accomplished (14). Much like through the use of checkpoint blockade therapy, if the signals that result in CD8 T cell exhaustion can be prevented through VEGF blockade, the CD8 T cells that infiltrate the tumor will be better able to kill tumor cells. Furthermore, these findings are not limited to just cancers of the CNS. Anti-angiogenic therapy is used in colorectal cancer and breast cancer treatment (15, 16). Likewise, immunotherapies are being tested in both of these types of cancer (17, 18). Therefore, our findings may be extrapolated to other combination strategies involving an immune therapy and anti-angiogenic therapy.

Here we show that anti-angiogenic therapy is not only a useful strategy to improve quality of life for patients diagnosed with GBM, but it may be a tractable approach to enhance immunotherapies. This study also builds upon our previous publication in which it was determined that combination therapy of picornavirus vaccination plus antiangiogenic treatment extended that lifespan of mice harboring GL261 gliomas (5). Therefore, we contend that we have identified another candidate for the family of checkpoint blockade treatments. VEGF blockade should be considered in pre-clinical models of immunotherapies to dually normalize the vasculature and enhance tumor antigen-specific CD8 T cell responses.

## Author contributions

CM, RK, KA, RI, KP, and AJ: Conceptualization; CM, RK, KA, and AJ: Formal analysis; AJ: Funding acquisition; CM, RK, KA, FJ, and AJ: Investigation; CM, RK, and AJ: Methodology; AJ: Project administration; JA, RI, and KP: Resources; AJ: Supervision; CM: Visualization; writing–original draft; CM, RK, KA, and AJ: Writing-reviewing and editing.

### Conflict of interest statement

The authors declare that the research was conducted in the absence of any commercial or financial relationships that could be construed as a potential conflict of interest. The handling Editor declared a shared affiliation, though no other collaboration, with the authors.
